# Adapted tissue assay for the assessment of ileal granulocyte degranulation following *in ovo* inoculation with select bacteria or coccidial challenge in chickens

**DOI:** 10.1371/journal.pone.0286532

**Published:** 2023-07-27

**Authors:** Audrey F. Duff, Kaylin M. Chasser, Kate E. McGovern, Michael Trombetta, Lisa R. Bielke

**Affiliations:** Department of Animal Sciences, The Ohio State University, Columbus, OH, United States of America; Foshan University, CHINA

## Abstract

A previously described heterophil degranulation assay was adapted for use with ileal mucosal tissue via quantification of β-_D_-glucuronidase and assay end product 4-methylumbelliferone (4-MU). Three initial experiments evaluated the effect of *in ovo* inoculations of *Citrobacter freundii* (CF) or mixed lactic acid bacteria (LAB) on ileal granulocyte degranulation. Inoculations were administered on embryonic d18, body weights (BW) were recorded on day of hatch (DOH) and d10 to calculate body weight gain (BWG), and ileal mucosal scrapings were collected on DOH or d10 for the 4-MU assay. In all experiments, treatments were statistically analyzed relative to control groups. Treatments minimally affected BWG in all *in ovo* experiments (*p* > 0.05) relative to respective control groups. Similarly, ileal degranulation in *in ovo* treatments did not statistically differ (*p* > 0.05). Based on BWG, *in ovo* treatments may have induced low-level inflammation unable to elicit detectable changes via the 4-MU assay. Four subsequent experiments were conducted to evaluate effects of *Eimeria maxima* (EM) on ileal degranulation. Treatments included non-inoculated controls and low, medium, or high EM infection. Across all four experiments, final BW or BWG over the inoculation period were suppressed (*p* < 0.05) in EM groups relative to respective controls with the exception of EM-low (*p* = 0.094) and EM-medium (*p* = 0.096) in one trial. Ileal mucosal scrapings for the 4-MU assay were collected on day of peak lesions. Resulting values were reduced (*p* < 0.05) for EM treated birds in three experiments with the exception of EM-medium (*p* = 0.247). No differences were observed in one experiment (*p* = 0.351), which may have been attributed to a variation in strain of infecting *Eimeria*. Although refinement for low level inflammation is warranted, results indicate successful adaptation of the 4-MU assay for use with intestinal tissue during significant gastrointestinal inflammation.

## Introduction

The gastrointestinal tract (GIT) of poultry has immediate and essential roles in poultry health and development from the time of hatch until the time of harvest. Primary roles include nutrient utilization, mucosal immune responses, serving as a reservoir for resident microbial communities, and formation of a protective barrier between GIT luminal contents and systemic circulation [1]. Direct insult to the GIT barrier and instances of barrier dysfunction can predispose birds to opportunistic diseases, nutrient malabsorption, and trigger a state of inflammation [2, 3]. Cumulatively, these conditions dampen overall health and performance that manifest in poultry as suppressed body weight (BW), body weight gain (BWG), and feed intake which collectively result in worsened feed conversion ratios and loss of profitability [4]. While not comprehensive, documented sources of inflammation and dysregulated barrier function in poultry include infection with opportunistic pathogens including enteropathogenic *E*. *coli*, *Campylobacter jejuni*, *Clostridium perfringens*, and *Salmonella* spp. as reviewed by Awad and coauthors [5], chronic stressors such as feed withdrawal, heat stress, or prolonged exposure to glucocorticoids [6–8], and coccidial infection [reviewed by 9, 10]. Due to the persistence of these conditions in the poultry industry, techniques that can assess the inflammatory process would be of value in determining severity of inflammation, and by proxy, severity of infections, tissue damage, or other forms of stressors. Such assessments could also provide insight into mechanisms by which various stimuli induce or cause persisting inflammation, or potentially provide therapeutic or prophylactic treatment targets.

Inflammation represents a vital and intricately coordinated component of innate immunity required to protect mucosal tissue against prolonged damage or antigenic threats via recognition and clearance of foreign elements and damaged host cells [reviewed by 11]. The primary function of acute GIT inflammation is to resolve specific threats, such as pathogens, dietary antigens, or tissue damage mediators, and facilitate tissue repair and a return to physiological homeostasis [reviewed by 12]. Broadly speaking, the inflammatory response is orchestrated through pattern recognition receptors (PRR) such as toll-like receptors (TLR), C-type lectin receptors, and nod-like receptors that are present in various immunocompetent cells and tissues [13]. Upon recognition and binding of compatible ligands, or pathogen associated molecular patterns (MAMPs), PRR trigger production of various signaling molecules including proinflammatory cytokines, chemokines, and transcription factors, which consequently influence functions of various effector cells, tissues and organs as reviewed by Medzhitov [14]. Initiation of this signaling cascade induces vasodilation, increased expression of adhesion molecules, and recruitment and extravasation of various leukocytes including mast cells, monocytes, and heterophils to the site of inflammation [reviewed by 15, 16]. While inflammation is generally considered beneficial to the host, chronic inflammation induced by long-term infection, injury, or exposure to noxious substances can further damage host tissues, prolong diseased states, and be detrimental to overall health [reviewed by 17, 18].

One of the foremost innate responses to inflammation in poultry is activation of heterophils, comparable in role and function to mammalian neutrophils [19]. These polymorphonuclear cells are among the first and most abundantly recruited leukocytes upon activation of the proinflammatory signaling cascade resulting in massive heterophil infiltration at the site of inflammation within 30 minutes of initial signaling [20, 21]. Heterophil roles in immunity include phagocytosis of microbes and foreign particles, production of reactive oxygen species, and release of antimicrobial compounds and enzymes via degranulation [22–24]. In contrast to neutrophils, heterophils generate a much weaker respiratory burst due to the absence of myeloperoxidase within their granules [25]. Instead, these granulocytes rely on highly effective phagocytosis, heterophil extracellular traps (HET), and degranulation components for proinflammatory debris clearance and resolution of infection [26]. Due to prominent roles in inflammation, quantification of heterophil activity, such as degranulation, could prove to be a useful index for assessment of the inflammatory response.

Myeloperoxidase has commonly been measured to assess neutrophil degranulation in mammalian studies, but is not a viable option in avian species [25, 27]. However, β-_D_-glucuronidase, an enzyme defined in heterophil granules, can serve as a suitable substitute in avian models [23, 27, 28]. Previously demonstrated assessment of heterophil degranulation has quantified β-_D_-glucuronidase in the supernatant of isolated peripheral blood heterophils stimulated with various bacteria [23, 28]. Here, a modified version of this assay for assessment of degranulation in intestinal mucosal tissue is reported. Notably, degranulation quantified in the modified assay is not heterophil specific as β-_D_-glucuronidase is present in the granules of mast cells [29], basophils [30], and eosinophils [31], and therefore represents an assessment of granulocyte degranulation. However, by using intestinal mucosa, additional site-specificity and real-time perspective of degranulation events in the GIT can be interpreted. The modified assay was evaluated in seven different experiments highlighting two models of inflammation. Experiments 1–3 assessed degranulation in 10 day-old chicks after *in ovo* inoculation with select bacterial inoculums previously demonstrated to influence immune and inflammatory responses in young chicks [32, 33]. Experiments 4–7 subjected birds to coccidial infection with *Eimeria maxima* (EM) and assessed degranulation on the day of expected peak *Eimeria* lesions.

## Materials and methods

### Animal housing and experimental design

A total of seven experiments were completed at the Poultry Research Facility at Ohio Agricultural Research and Development Center (OARDC) in Wooster, Ohio. Experiments were carried out under approved animal care protocols per The Ohio State University Institutional Animal Care and Use Committee: experiments 1–3 #2016A00000038; experiments 4 and 5 #2019A00000015; and experiments 6 and 7 #2016A00000041. In experiments 1–3 and 5, eggs were obtained from a local hatchery (Ross 708) or on-site flock (Leghorn), respectively, and hatched at the OARDC Poultry Research Facility. In experiments 4, 6, and 7, day of hatch (DOH) Ross 708 chicks were obtained from a local hatchery. On DOH in all experiments, chicks were neck tagged according to treatment and reared on wire floor cages (Exp. 1–5 and 7) or floor pens with fresh pine shavings (Exp. 6) for the duration of the experiments. All birds were provided *ad libitum* access to water and diets formulated to meet nutritional requirements [34], and ambient light was maintained within age appropriate ranges. At the end of experiments 1–5, birds were euthanized by CO_2_ inhalation for sample collection, as described below, while birds in experiments 6 and 7 were euthanized by cervical dislocation.

### Embryo inoculation, incubation, and hatching—experiments 1–3

Ross 708 eggs were obtained from a local hatchery and placed in a single stage incubator (Natureform Inc. Jacksonville, FL) at the OARDC Poultry Research Facility until embryonic day 18 (ed18). At this time, eggs were candled to confirm fertility and prepped for *in ovo* inoculation. Iodine (Povidone-Iodine 10% topical solution, Drug Mart, Wooster, OH) was used to sterilize the air-cell end of each egg, and a small hole was punched aseptically into the shell to administer *in ovo* inoculums using a partially sheathed needle to control depth of injection. Treatments were injected into the amnion and consisted of 200μL of 0.9% sterile saline (S), or approximately 1x10^2^ CFU/egg of *Citrobacter freundii* 97A4 (CF) originally isolated from the GIT of healthy chickens [35, 36], or previously [37] described mixed lactic acid bacteria (LAB) which consisted of *Lactobacillus salivarius* and *Pediococcus* spp. (L). Selection of CF and LAB inoculums was based on microbiome and proteomics work previously described by Wilson et al. [33, 36] and Rodrigues et al. [32, 38]. After inoculation, eggs were allocated into two separate benchtop hatchers (Hova-Bator model 1602N, Savannah, GA, United States) per treatment, for a total of six hatchers. Each hatcher contained either 15 (Exp. 1), 17 (Exp. 2) or 21 (Exp. 3) eggs per replicate hatcher depending on fertility rates and desired sample size. All hatchers were disinfected with a 10-fold diluted bleach solution prior to use and equipped with digital thermometers capable of measuring temperature and humidity to mimic incubation conditions in commercial settings [39]. Incubation conditions were recorded three times daily from ed18 until hatch. On DOH, an even distribution of chicks from each replicate incubator were comingled in wire floor cages according to treatment in order to mitigate hatcher effects and variability (Table 1).

**Table 1:** Experimental design for experiments 1–3.

Exp.	Trt	Pen n	Replicates	ed18 Dose (CFU/embryo)	Sample Time	Sample n/Trt^1^
1	S1	10	2	1x10^2^	d10	6*
	CF1	10	2			
	L1	10	2			
2	S2	10	2	1x10^2^	d10	
	CF2	10	2			
	L2	10	2			
3	S3	10	2	1x10^2^	DOH and d10	
	CF3	10	2			
	L3	10	2			

All embryos were aseptically inoculated on embryonic d18 with 200μL of Saline, *Citrobacter freundii* 97A4, or a mixed LAB inoculum via *in ovo* injection. Each wire floor cage treatment replicate was composed of five chicks from each treatment replicate hatcher for a total of 10 chicks per pen.

S: Saline; CF: *Citrobacter freundii* 97A4; L: mixed lactic acid bacteria; ed18: embryonic day 18; DOH: day of hatch; Trt: treatment

^1^3 chicks sampled per replicate pen for a total of 6 chicks per treatment.

*6 birds sampled on both DOH and d10 in Exp. 3.

### *In ovo* bacterial preparation—experiments 1–3

Bacterial inoculums were prepared as described by Wilson and coauthors [36]. An aliquot of each selected isolate was inoculated into tryptic soy broth (Sigma-Aldrich, St. Louis, MO) at ~1.5% volume and incubated aerobically at 37°C overnight. After incubation, cells were washed three times in sterile 0.9% saline by centrifugation at 1,800 × *g* for 15min. Approximate culture cell concentrations were spectrophotometrically quantified (Spectronic 2000, Thermo Scientific, Waltham, MA) and serial diluted to reach the desired inoculum concentration. Inoculum concentrations were later retrospectively confirmed by serial dilution plating on tryptic soy agar and overnight aerobic incubation at 37°C. Confirmed CF and LAB inoculums administered *in ovo* were, respectively, 6.6x10^2^ and 3.34x10^2^ (Exp. 1); 6.67x10^2^ and 5.87x10^2^ (Exp. 2); and 5.07x10^2^ and 5.73x10^2^ (Exp. 3) CFU/egg.

### *Eimeria* preparation and inoculation—experiments 4–7

A pure culture of *E*. *maxima* Guelph (EMG; Exp. 4), mixed culture of EMG and *E*. *acervulina* (EMG+A; Exp. 5), or pure culture of *E*. *maxima* M6 (EMM; Exp. 6 and 7) were used to induce coccidiosis in experiments 4–7. Prior to oral challenge, inoculum stocks were floated in a saturated NaCl solution (1:10; v:v), quantified with a McMaster’s chamber, and calculated as number of sporulated, infective, oocysts per mL of stock solution. Oocysts were then resuspended to the desired inoculum concentration with sterile 0.9% saline. In Exp. 4–6, treatments included a non-inoculated *Eimeria*-free control (C) and a single *Eimeria* treated group (EM), while Exp. 7 consisted of a non-inoculated control and three *Eimeria* treated groups of different inoculation concentrations (Table 2). Treatment size, *Eimeria* inoculum concentration, and timing of inoculum administration are varied between experiments 4–7 as some of the birds sampled in these experiments were part of larger, unrelated coccidia trials. However, timing of sample collection was consistent in that samples were taken on day of peak *E*. *maxima* lesions when the most significant coccidial-related GIT insult was expected.

**Table 2:** Experimental design for experiments 4–7.

Exp.	Trt	Trt n^1^	*Eimeria* spp. Challenge	Dose (oocysts/bird)	Inoculation Time	Sample Time	Sample n/Trt^2^
4	EM4	10	EMG	5x10^3^	d20-d23*	d28	6
5	EM5	10	EMG+A	2x10^4^	d11	d16	6
6	EM6	20	EMM	1x10^4^	d16	d22	10
7	EM-low	10	EMM	5x10^3^	d9	d14	6
	EM-medium	10		1x10^4^			6
	EM-high	10		1.5x10^4^			6

Various *E*. *maxima* or *E*. *maxima* and *E*. *acervulina* challenge inoculums were administered to induce coccidiosis. All inoculums were administered via oral gavage, and samples were collected on the day of peak *E*. *maxima* lesions. Each experiment consisted of an *Eimeria* challenged group, described below, and a non-inoculated, *Eimeria*-free control of equal size (C4, C5, C6, C7).

EMG: *E*. *maxima* Guelph; EMG+A: *E*. *maxima* Guelph and *E*. *acervulina*; EMM: *E*. *maxima* M6; Trt: treatment

^1^Each treatment consisted of 1 replicate with the exception of Exp. 6 which consisted of 10 replicate pens/trt of 10 birds each from which only 1 bird per pen was sampled.

^2^Sample n/treatment was the same for all corresponding experimental controls.

*Birds in Exp. 4 were dosed for 4 consecutive days.

### Sample collection

#### Experiments 1–3

Chicks in each *in ovo* inoculation experiment were reared in wire floor pens with 10 chicks/pen (20 chicks/treatment) and were sampled on DOH (Exp. 3 only; n = 3/replicate pen) and d10 (n = 3/replicate pen). In experiments 1 and 2, treatments consisted of S1, CF1, and L1 or S2, CF2, and L2, respectively. For experiment 3, treatments included S3_h_, CF3_h_, and L3_h_ and S3_10_, CF3_10_, and L3_10_ for DOH and d10 sampled chicks, respectively. On DOH and d10, BW were measured for all birds in experiments 1–3 in order to calculate BWG. For sample collection, a section of the lower ileum, distal to Meckel’s Diverticulum, was opened longitudinally and gently cleared of digesta with the blunt side of a razor blade. The razor blade was then used to collect approximately 0.2–0.5g of ileal mucosal scrapings into 2.0mL centrifuge tubes (Fisher Scientific, Waltham, MA) filled with a small volume of RPMI 1640 media (Catalog #MT10041CV, Fisher Scientific, Waltham, MA) supplemented with 1% penicillin/streptomycin (Catalog #P0781; Sigma Aldrich, St. Louis, MO), hereon referred to as RPMI+1%ps, and kept on ice.

#### Experiment 4

Birds used in experiment 4 were part of a larger scale *E*. *maxima* propagation experiment. For the purposes of experiment 4, a subset of 20 Ross 708 broilers were designated as the experimental n and assigned to either a non-inoculated control group (C4; n = 10) or an EMG inoculated group (EM4; n = 10). All C4 birds were fed an anticoccidial starter diet (Bio-cox60, Huvepharma Inc., Peachtree City, GA) in order to minimize the risk of coccidial contamination, while EM4 were fed a basal starter diet that met nutrition requirements. From d20-d23, EM4 received daily inoculations of 5x10^3^ EMG oocysts/bird via oral gavage (OG). On d28, final BW was measured on all birds and sample collection (n = 6/treatment) occurred as described for experiments 1–3. Samples were collected at 5 days post inoculation of the final consecutive EMG dose, in order to measure coccidial impact on the day of peak lesions (Table 2).

#### Experiment 5

Experiment 5 used Leghorn chicks that were hatched at the OARDC Poultry Research Facility and were randomly allocated to a non-inoculated control (C5; n = 10) or EMG+A inoculated treatment (EM5; n = 10) one replicate pen each. As in experiment 4, C5 birds were kept on an anticoccidial diet for the duration of the study. Birds in EM5 were administered 2x10^4^ EMG+A oocysts/bird via OG on d11, at which time all birds from C5 and EM5 were individually weighed. On d16, day of peak lesions, BW measurements were collected to assess BWG, and ileal mucosal scrapings were collected from 6 birds/treatment (Table 2).

#### Experiment 6

Experiment 6 samples were also collected from birds that were part of a larger, unrelated *Eimeria* trial. Here, the subset of Ross 708 broilers sampled represented a non-inoculated control (C6) and an EMM inoculated treatment (EM6). On d16, EM6 birds were inoculated with 1x10^4^ EMM oocysts/bird via OG. On d22, expected peak lesions, C6 and EM6 birds were individually weighed to determine BW, and ileal mucosal scrapings were collected. The larger experiment consisted of 10 replicate pens with 10 birds each, but during sample collection for experiment 6, only 1 bird per replicate C6 or EM6 pen was sampled (n = 10 samples/treatment). However, BW was assessed on a total of 20 birds/treatment due to simultaneous sampling occurring in the original experiment (Table 2). Here, BWG could not be calculated as the original experiment design called for weekly pen weights, with individual weights only to be assessed on the concurrent sampling day.

#### Experiment 7

A total of 40 Ross 708 broiler chicks were randomly assigned to treatments (n = 10/treatment) that included a non-inoculated control (C4), low EMM inoculation (EM-low), medium EMM inoculation (EM-medium), or high EMM inoculation (EM-high). On d9, all birds were weighed, and EM-low, EM-medium, and EM-high were inoculated with 5x10^3^, 1x10^4^, or 1.5x10^4^ EMM oocysts/bird, respectively, via OG. On d14 all birds were reweighed to calculate BWG, and ileal mucosal scrapings were collected from 6 birds/treatment (Table 2). In experiment 7, samples were taken one day prior to when peak lesions were expected due to miscalculated sample timing.

### Degranulation assay

Granulocyte degranulation was assessed in ileal mucosal scrapings by quantifying β-_D_-glucuronidase fluorometrically with a modified protocol previously described for use with poultry heterophils [23, 27, 28]. In contrast to measuring β-_D_-glucuronidase released in peripheral blood heterophil cell culture supernatant, the assay described here used ileal mucosal scrapings from chickens exposed to inflammatory stimuli as the origin of stimulated heterophils. Reagents were identical to original descriptions of this assay [27], with minor modifications to sample collection and handling. As previously mentioned, 0.2–0.5g of mucosal scrapings were collected into 2.0mL centrifuge tubes preloaded with a small volume of RPMI+1%ps on ice in order to preserve tissue integrity and limit bacterial production of β-_D_-glucuronidase [40, 41]. Healthy lung tissue from a control bird was collected and treated in a similar fashion in order to serve as a negative control due to low amount of expected degranulation, and additional ileal mucosal scrapings were collected from a control bird to serve as a positive control, described below. After collections, samples were reconstituted at 1:2 (w:v) in RPMI+1%ps and gently homogenized with a sterile, blunted Pasteur pipette taking care not to cause cell lysis and subsequent false positive degranulation values. Samples, including the negative control but not positive, were then centrifuged at 20,000 × *g* for 5min to thoroughly pellet mucosal tissue and supernatants were collected. The positive control sample was more thoroughly homogenized using the Pasteur pipette so that the resulting suspension included small amounts of tissue. To generate a true positive control, serum opsonized zymosan (8mg/mL Exp. 5, or 32mg/mL Exp. 6 and 7) was added as an agonist at 1:1 (v:v) with the positive control suspension, and an equivalent volume of RPMI+1%ps was added to sample supernatants, including the negative control. Samples were loaded into 0.2mL snap cap tubes (VWR, Radnor, PA) and collectively incubated at 42°C for 1h, after which all samples were centrifuged at 2,000 × *g* for 5 minutes to recollect supernatants. No positive controls were included in the first several iterations of this assay (Exp. 1–4). Lastly, all sample supernatants, including controls, were diluted either 100-fold (Exp. 1–4) or 1,000-fold (Exp. 5–7) in RPMI+1%ps to keep fluorescence within the readable range of the plate reader and assay standard curve. Sample supernatants may be stored overnight, but storage in excess of 24h will yield inadequate results.

Prepared sample supernatants and standards (25μL) were added in triplicate in a black, non-treated, flat-bottom 96-well plate (VWR, Radnor, PA) and incubated with 50μL of freshly prepared substrate (10mM 4-methylumbelliferyl-β-_D_-glucuronide (4-MUG; Catalog #50-493-344; Fisher Scientific, Waltham, MA), 0.1% Triton X-100 in 0.1M sodium acetate buffer) at 42°C for 4h in the dark. The assay standard consisted of 4-methylumbelliferone (4-MU; Catalog #5980-33-6, Sigma-Aldrich, St. Louis, MO) concentrations ranging from 200μM 4-MU to 0μM that decreased by 2-fold increments. After assay incubation, the reaction was stopped with the addition of a stop solution (0.05M glycine and 5mM EDTA; pH 10.4) and liberated 4-MU was quantified fluorometrically (Synergy HTX, multi-mode microplate reader, BioTek Instruments Inc., Winooski, VT, USA) at excitation/emission wavelengths 360/460nm. In experiments 1–4 the gain setting on the plate reader was set to auto-gain, whereas in experiments 5–7, this setting was adjusted to a gain of 70. After subtraction of the negative control raw fluorescent value from samples to account for autofluorescence, sample values were converted to nanomoles of 4-MU using the standard curve of known concentrations and retrospectively corrected for dilutions that occurred during sample preparation.

### Statistical analysis

Mean and standard error values were calculated for BW, BWG, and liberated 4-MU values for each treatment. Data from experiments 1–3 and 7 were subject an Analysis of Variance as a completely randomized design in JMP Pro 15 [42], and differences amongst means was determined using Dunnett’s post-hoc analysis which compared challenged treatments against respective control groups. Experiments 4–6 were analyzed using a t-test. Significance was determined at *p* < 0.05 in all experiments, and all data are represented as mean ± standard error.

## Results

### Body weight and body weight gain

**Fig 1:**
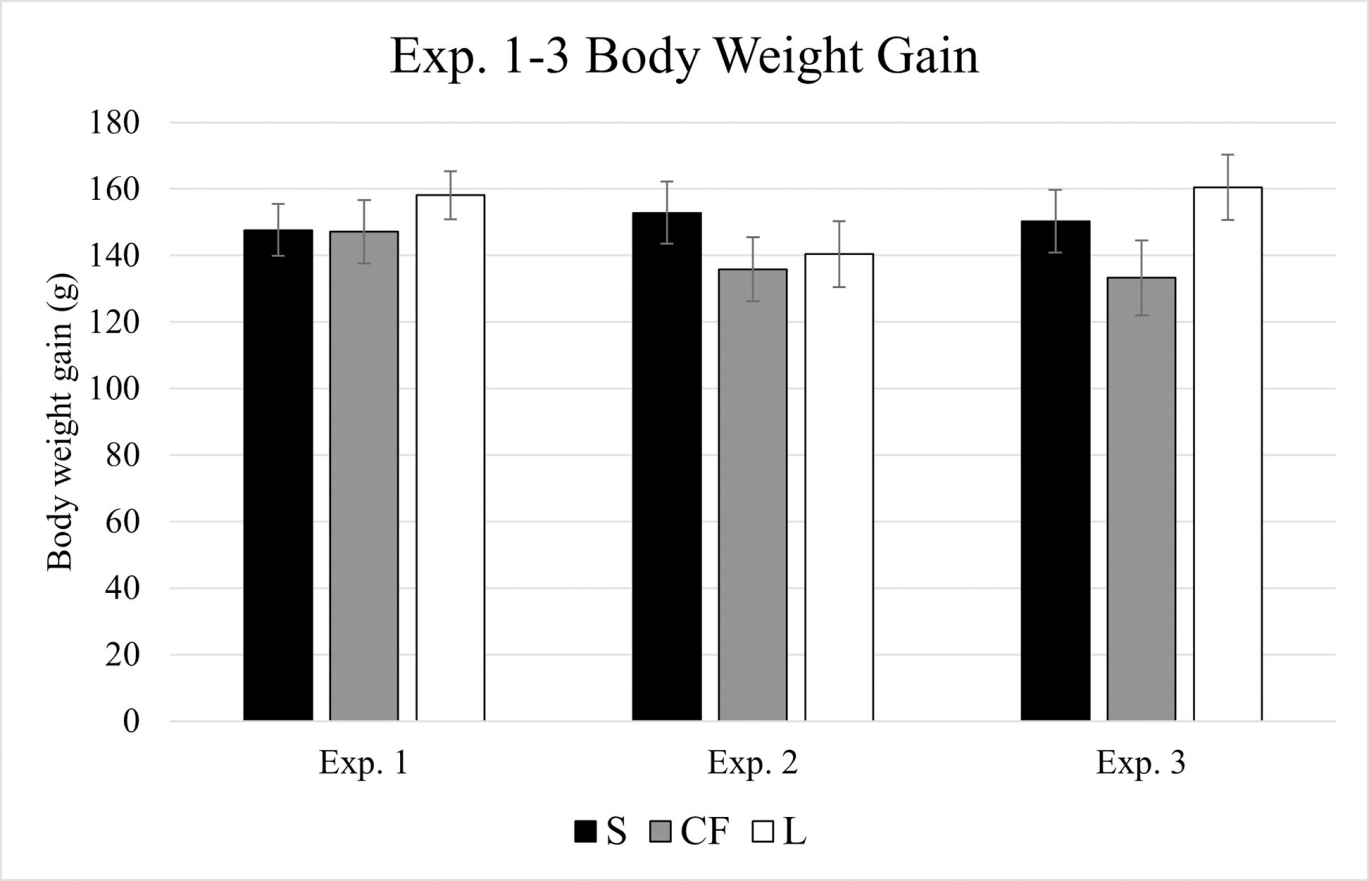
Body weight gain for experiments 1–3. All chicks received *in ovo* inoculations on ed18 consisting of saline or 10^2^ cells of *C*. *freundii* or LAB inoculums. Body weights were measured on day of hatch and d10 to calculate body weight gain over the experimental period. Data are presented as mean ± standard error (n = 20 chicks/treatment). Data were analyzed via ANOVA and Dunnett’s post-hoc test. No significant differences (*p* > 0.05). S: Saline; CF: *Citrobacter freundii* 97A4; L: mixed lactic acid bacteria.

**Fig 2:**
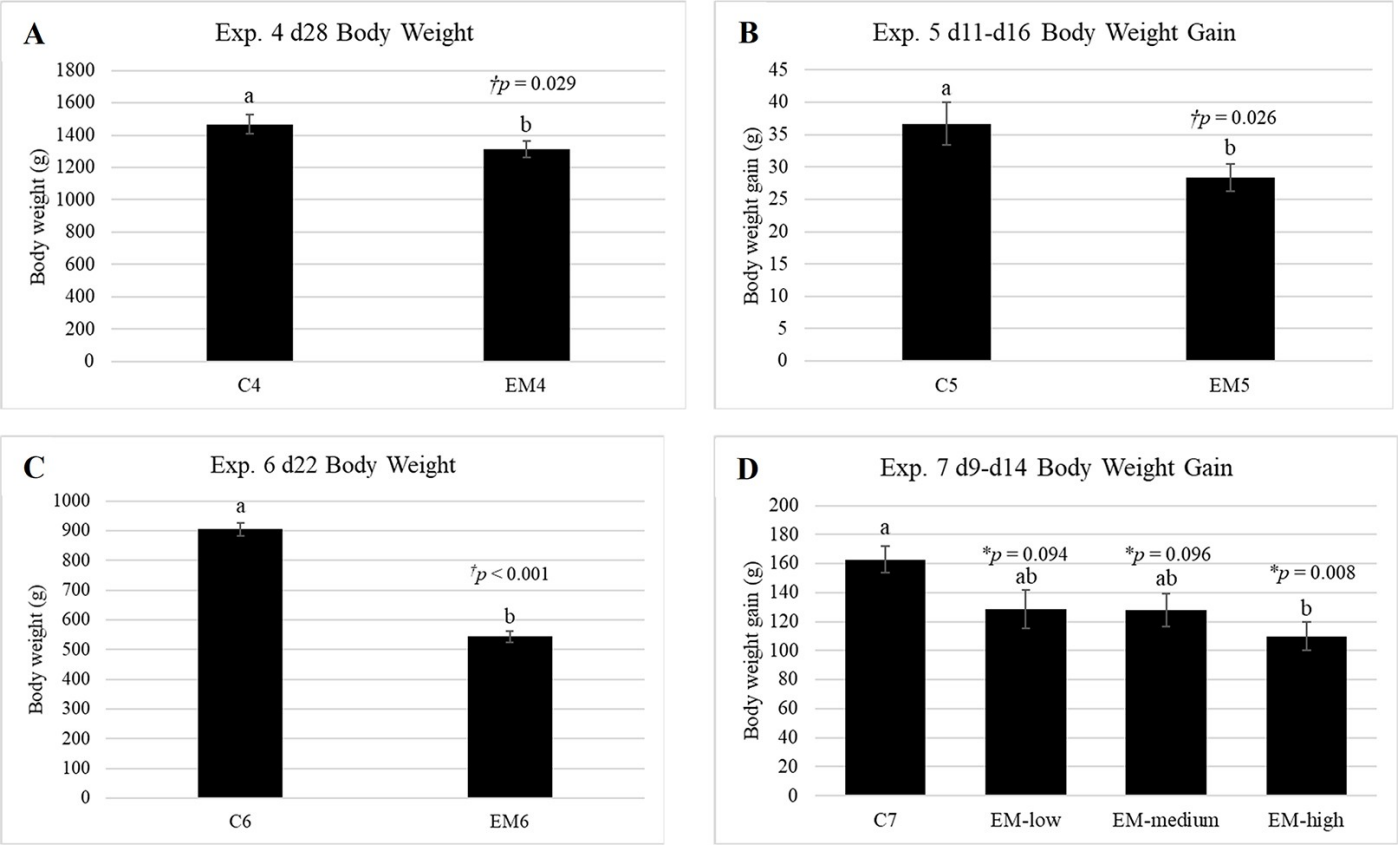
Body weight and body weight gain for experiments 4–7. Final body weight was assessed on the day of peak *Eimeria maxima* lesions in Exp. 4 and 6. Body weight gain was assessed over the *Eimeria* inoculation period (time of inoculation to time of peak shedding) in Exp. 5 and 7. Data are presented as mean ± standard error (n_Exp. 4, 5, 7_ = 10 birds/treatment; n_Exp. 6_ = 20 birds/treatment). ^†^Data analyzed via t-test. *Data analyzed via ANOVA and Dunnett’s post-hoc test. ^a,b^Letters represent mean values with significant differences relative to the control treatment (*p* < 0.05).

Cumulative body weight gain data for experiments 1–3 is presented in Fig 1. No significant differences were observed for CF nor L treated birds relative to S in any of the three experiments (*p* > 0.05). Additionally, no consistent trends in BWG were observed for CF or L treated groups relative to S across experiments (Fig 1). In contrast, *Eimeria* inoculation had a noticeable suppressive effect on BW and BWG in all experiments (Fig 2A–2D). In experiment 4, EM4 was significantly (*p* = 0.029) lighter than C4, with an average BW 156g less than control birds (Fig 2A). Likewise, final BW of EM6 at time of peak lesions in experiment 6 was reduced by 361.25g (*p* < 0.001) relative to C6 (Fig 2C). When BWG was assessed over the *Eimeria* challenge period, time of initial *Eimeria* inoculation to peak lesions, EM5 BWG in experiment 5 was suppressed by an average of 10g (*p* = 0.026) relative to C5 (Fig 2B). Interestingly, while all EM groups exhibited numerically depressed BWG relative to C7 in experiment 7, only EM-high was significantly lower (*p* = 0.008) than C7 having gained an average of 53g less than control counterparts (Fig 2D).

### Ileal granulocyte degranulation

**Fig 3:**
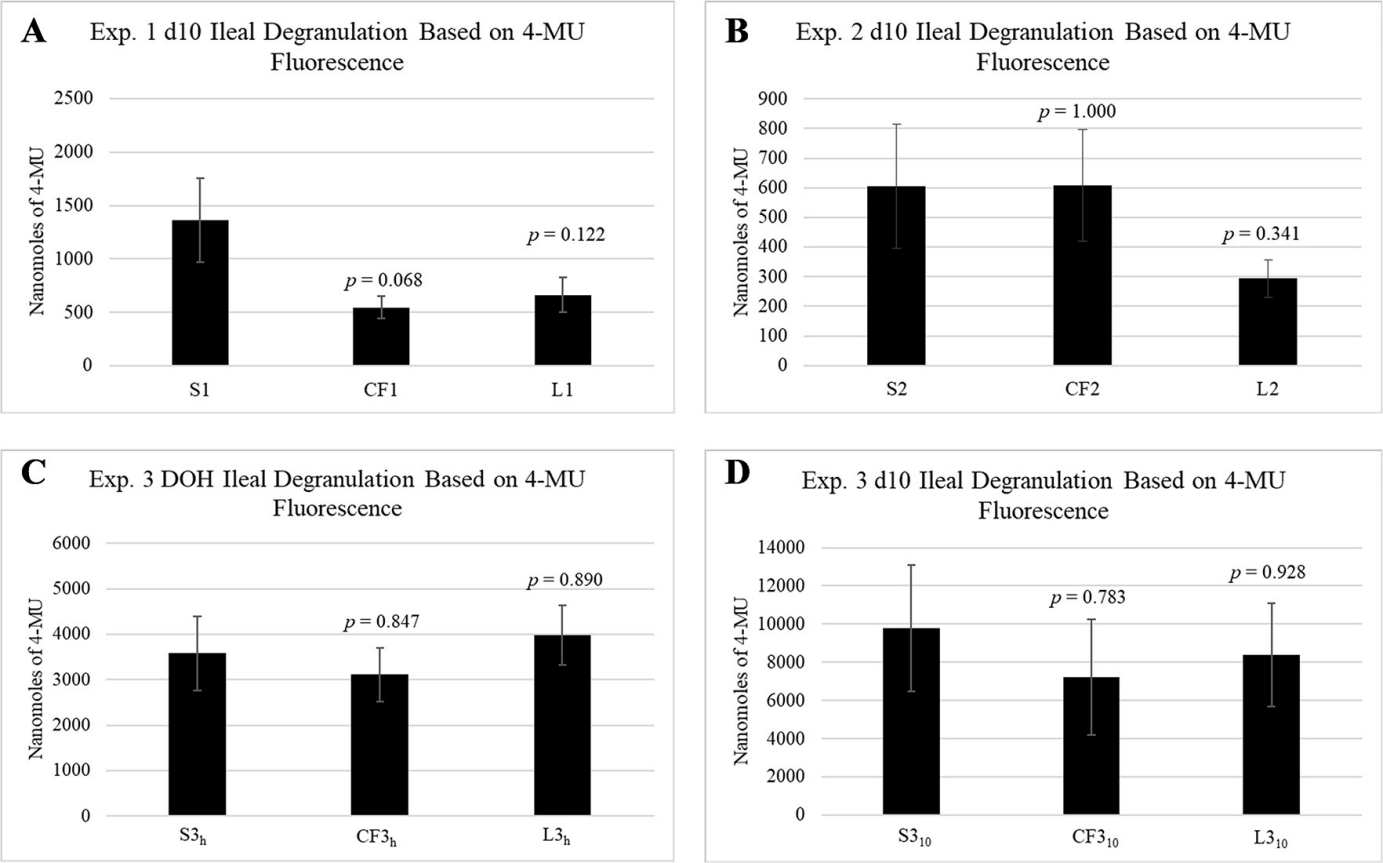
Ileal granulocyte degranulation for experiments 1–3. All chicks received *in ovo* inoculations on ed18 consisting of saline or 10^2^ cells of *C*. *freundii* or LAB inoculums. On day of hatch (Exp. 3 only) and d10, ileal segments were collected to assess ileal granulocyte degranulation via β-_D_-glucuronidase activity. Samples were incubated with 4-MUG at 42°C for 4h and liberated 4-MU was quantified as an indicator of degranulation. Data are presented as mean ± standard error (n = 6 chicks/treatment). Fluorescent plate reader gain setting: auto-gain. Data were analyzed via ANOVA and Dunnett’s post-hoc test. No significant differences (*p* > 0.05).

Degranulation activity was assessed by quantifying the interaction between β-_D_-glucuronidase and 4-MUG via subsequently liberated 4-MU. In experiments 1–3, there did not appear to be a treatment specific effect on degranulation (Fig 3). While CF1 and L1 4-MU values were numerically lower than S1, differences were insignificant (*p* > 0.05). However, CF1 was decreased at a significance of *p* = 0.068 (Fig 3A). In experiment 2, CF2 was almost equivalent in mean nanomoles of 4-MU relative to S2 (*p* = 1.000), and L2 showed no significant difference (*p* = 0.341). Degranulation activity in experiment 3 on DOH and d10 is depicted in Fig 3C and 3D. While overall degranulation activity appears to increase over time, as denoted by the change in y-axis scale, no differences were observed between CF3_h_ (*p* = 0.847) or L3_h_ (*p* = 0.890) relative to S3_h_ (Fig 3C), nor between CF3_10_ (*p* = 0.783) or L3_10_ (*p* = 0.928) relative to S3_10_ (Fig 3D). Notably, all degranulation results from experiments 1–3 exhibited a relatively high degree of error across treatments (Fig 3).

**Fig 4:**
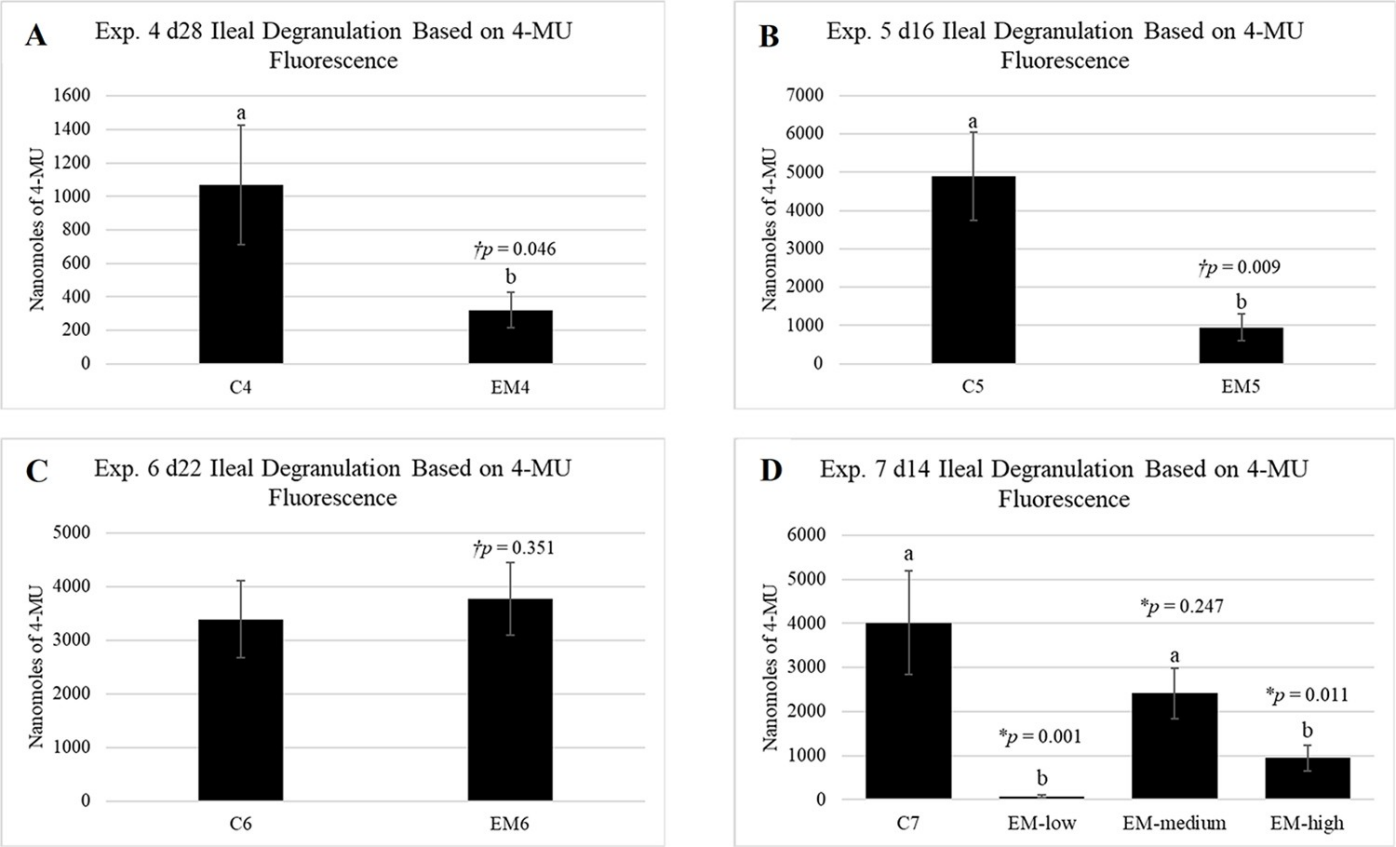
Ileal granulocyte degranulation for experiments 4–7. On the day of peak *Eimeria maxima* lesions, ileal segments were collected to assess ileal granulocyte degranulation via β-_D_-glucuronidase activity. Samples were incubated with 4-MUG at 42°C for 4h and liberated 4-MU was quantified as an indicator of degranulation. Data are presented as mean ± standard error (n_Exp. 4, 5, 7_ = 6 birds/treatment; n_Exp. 6_ = 10 birds/treatment). Fluorescent plate reader setting: auto-gain Exp. 4; gain 70 Exp. 5–7. ^†^Data analyzed via t-test. *Data analyzed via ANOVA and Dunnett’s post-hoc test. ^a,b^Letters represent mean values with significant differences relative to the control treatment (*p* < 0.05).

As observed with BW and BWG, degranulation trends were markedly different in experiments 4–7 relative to *in ovo* experiments, and coccidiosis seemed to have a dramatic, negative effect on quantifiable degranulation (Fig 4). In both experiments 4 and 5, EM4 and EM5 mean 4-MU values were significantly lower (*p* = 0.046 and *p* = 0.009, respectively) than corresponding controls, C4 and C5 (Fig 4A and 4B). However, in experiment 6, EM6 degranulation was similar to C6 (*p* = 0.351) with a slight numeric increase in relative mean value (Fig 4C). All *Eimeria* groups in experiment 7 presented numerically suppressed 4-MU values relative to C4. Curiously, only EM-low (*p* = 0.001) and EM-high (*p* = 0.011) had significantly less β-_D_-glucuronidase activity relative to C4, while EM-medium (*p* = 0.247) did not statistically differ (Fig 4D). Similar to experiments 1–3 (Fig 3), there was a high degree of error observed within 4-MU values in experiments 4–7, however, there appeared to be more variability in control groups as opposed to EM groups, with the exception of EM6 and EM-medium (Fig 4). Positive controls were included in 4-MU assays completed for experiments 5–7.

## Discussion

Colonization of the microbiota has been strongly implicated in the development of gut associated lymphoid tissue and immune function, while pathogenic infection or persistent exposure to noxious substances has been shown to induce acute or chronic inflammation and subsequent tissue damage [reviewed by 43, 44]. A total of ten TLR have been described in the chicken that are differentially distributed on multiple innate immune cells including natural killer cells, dendritic cells, epithelial cells, heterophils, and macrophages and facilitate the recognition of various microbial MAMPs [reviewed by 45, 46]. Binding of TLR agonists is known to stimulate transcription of proinflammatory mediators and effector functions in these cells [reviewed by 47]. The stimulatory effect of TLR recognition of microbial MAMPs on heterophil degranulation in the chicken has been well demonstrated [23, 28, 48]. However, heterophil degranulation during coccidial infection is a lesser documented phenomenon [49, 50]. These series of experiments demonstrated the ability to adapt a previously described heterophil degranulation assay [23, 27] for use with intestinal mucosal tissue. However, noticeably different, and unexpected, results were produced for the two different models of inflammation used which included manipulation of pioneer colonization and coccidiosis, which highlights the fact that inflammation pathways are variable and thus, there is a strong need for multiple assays to characterize gastrointestinal inflammation.

In experiments 1–3, chick embryos were inoculated with bacterial isolates previously demonstrated to alter immune function and cellular responsiveness to inflammation as represented by differentially expressed proteins (DEP) and associated Z-scores [32, 33]. In the work of Rodrigues and coauthors [32], chicks were inoculated with S, CF, or LAB as described for experiments 1–3, and ileal samples were collected at 10 days-of-age for protein extraction and input into Ingenuity Pathway Analysis (IPA) to assess the impact of pioneer colonization on intestinal protein expression. Predictive function analysis in IPA revealed DEP associated with *in ovo* inoculums caused differential annotation of inflammatory signaling in broiler chicks. Chicks inoculated with CF had a predicted high activation Z-score concomitant with inflammation of organ, but negative functional scores associated with inhibition of inflammatory response and cell movement of granulocytes and leukocytes relative to S. In contrast, LAB inoculated chicks exhibited an activation of DEP associated with inflammation of organ relative to S, but also an increased responsiveness to inflammation. Cellular functions associated with leukocyte migration, immune response of leukocytes, cellular infiltration by leukocytes, movement of leukocytes and granulocytes and inflammatory response were activated in LAB chicks relative to CF [32]. Collectively, these results demonstrated an influence of pioneer colonization on reprogramming overall onset of inflammatory status and responsiveness to inflammation.

Based on IPA predicted function of leukocytes and granulocytes [32], it was hypothesized that CF groups in experiments 1–3 would have suppressed 4-MU degranulation values relative to S and LAB, and that LAB would exhibit increased degranulation relative to CF and potentially S. However, no significant differences were observed between any of the *in ovo* treatments reported (Fig 3). Similarly, BWG between S, CF, and LAB treatments was insignificant in all three *in ovo* trials (Fig 1). Suppressed body weight is a common indicator of inflammation in broilers as nutrients are diverted away from growth and redistributed to fuel immune responses such as the APR, and immune functions such as cell signaling, differentiation and migration [51, 52; reviewed by 53]. Therefore, it is feasible to suspect that *in ovo* bacterial inoculums in experiments 1–3 did not elicit a strong enough inflammatory response to produce changes in degranulation within detectable limits of the 4-MU assay. Furthermore, bacterial isolates selected for experiments 1–3 were not of pathogenic origin, which was an important experimental design difference between the modified 4-MU assay and original assay which stimulated heterophils with *Salmonella* [23]. The CF strain was isolated from the ceca of healthy adult chickens [35] while the LAB inoculum consisted of beneficial probiotic bacterial strains [33, 37]. Therefore, perhaps degranulation was more consistent with commensal bacterial colonization resulting in statistical similarity to experiment 1–3 controls. Furthermore, sample collection on d10 may have been delayed with regard to immune effects elicited by *in ovo* inoculation. These results also highlight the challenges of replicating *in silico* data with *in vivo* models and parameters.

Infection with *Eimeria* spp. elicits in a significant degree of inflammation within the GIT due to multiple cycles of epithelial invasion and damage observed over the course of the parasite’s reproductive phases [54, 55]. The intra-host lifecycle of *Eimeria* is comprised of intracellular, extracellular, asexual and sexual stages of reproduction which stimulate a comparably complex immune response as reviewed by Lillehoj [56]. It is well documented that both antibody and cell-mediated immune responses ensue subsequent to coccidial infection, although T-cell mediated responses are the predominant driving force in development of long-term protective immunity [57; reviewed by 56, 58, 59]. Nevertheless, innate recognition of conserved antigens is the first line of defense during coccidial infection [reviewed by 60]. In poultry, MAMPs and TLR associated with coccidial infection are somewhat poorly defined [61] but may include TLR ligands such as profilin or microneme proteins involved in parasite adhesion and invasion [62–64]. As previously highlighted, recognition of agonistic TLR ligands stimulates various effector functions in innate immune cells, including heterophils [48]. However, limited literature is available examining in depth roles of heterophils during coccidial infection [49, 50, 57, 65].

In experiments 4–7, coccidial infection was confirmed by observation of significantly suppressed BW and BWG (Fig 2) as well as petechial hemorrhages in the intestine observed during sampling. Relative to other chicken *Eimeria* species, *E*. *maxima* is highly immunogenic and a small number of oocysts are able to generate a robust immune response [66]. Therefore, it was hypothesized that EM groups would exhibit measurable changes in degranulation relative to healthy controls. In a study by Wei et al. [50], authors found that isolated chicken heterophils stimulated 1:1 with *E*. *tenella* sporozoites *in vitro* resulted in the release of HETs, a finding that was also verified in neutrophils stimulated with various *Eimeria* species in bovine and caprine models [67, 68]. Similarly, Zhou and coauthors [65] found that live and heat-killed *E*. *tenella* oocysts stimulated expression of chicken (ch)TLR4, chTLR15, and MyD88 in chicken heterophils which would be suspected to lead to heterophil effector functions. Surprisingly, EM coccidial infection resulted in significantly lower degranulation in almost all *Eimeria* treated birds in experiments 4–7 relative to respective controls (Fig 4). This decreased β-_D_-glucuronidase release, and by proxy degranulation, in heterophils during *E*. *maxima* infection is a newly reported finding. While in conflict with previously highlighted literature, several possibilities exist which may explain these differences in results. With respect to the findings of Wei et al. [50], perhaps degranulation and release of HETs represent different phenomena. More specifically, chicken HETs have been described as extracellular netlike structures composed of DNA, histone-DNA complex, and elastase from heterophil granules [69]. Therefore, it is possible that HETs do not contain high concentrations of β-_D_-glucuronidase which would not only set degranulation and HET release apart, but also explain why 4-MU results did not reflect a similar trend in heterophil activity. However, this hypothesis may be unlikely. Additionally, 4-MU samples were only assessed at time of peak EM lesions. Rose et al. [49] reported a biphasic increase in heterophils before and after peak oocysts shedding, with marked changes in circulating heterophils within 3 hours of infection [49, 57], but such timing was not feasible for these studies. Based on these findings, it is possible quantifiable degranulation was not occurring at time of peak lesions and oocyst shedding and that degranulation events were missed in experiments 4–7. Therefore, observation of degranulation trends as a response of coccidial infection over time would provide further clarity.

Lastly, and most likely, suppressed degranulation in experiments 4–7 could be a product of morphological changes in the GIT caused by coccidial infection. Mucosal sloughing is commonly associated with *Eimeria* infection [70, 71], therefore the authors posit the possibility that decreased degranulation values were the result of fewer heterophils present in the mucosa at the time of sampling due to tissue sloughing. While the exact mechanism behind observed degranulation is not known at this time, it is worth noting that EM groups tended to exhibit a lower degree of variability in the 4-MU assay relative to controls (Fig 4). It is possible that this is because, relative to controls, the health status of EM birds was more consistent due to uniformity of coccidial infection. The alternative may be true in EM6 and EM-medium (Fig 4C and 4D, respectively) where a higher degree of error was observed, potentially indicating a less uniform infection which may have lent to the lack of significant difference relative to C6 and C4, respectively. The lack of significance associated with experiment 6 4-MU values could also possibly be attributed to a variation in the infecting strain of *Eimeria*. Furthermore, discrepancies observed in 4-MU dose-response to *Eimeria* in experiment 7 may be related to efficiency of coccidial infection [72]. In this instance, perhaps the EM-low dose represented a more realistic and highly efficient infection, while the EM-medium dose resulted in less efficient infection, possible overcrowding of oocysts in the intestine, less efficient reproductive life cycles, and less intestinal damage and measurable effects on degranulation. In contrast, EM-high may have been a high enough dose to overcome efficiency of infection due to sheer number of infective oocysts and thus caused excessive physiologic damage like mucosal sloughing or impaired GIT morphology as observed in graded *Eimeria* infections reported by Teng et al. [73]. This may have resulted in lower 4-MU values due to a reduced amount of mucosa and therefore, recoverable amount of tissue scrapings and granulocytes. To further improve upon the data obtained in the coccidial experiments, future iterations of the 4-MU assay would correlate 4-MU values, BWG, and *Eimeria* lesions scores [74] on a per bird basis in order to elucidate whether disease severity has an impact on degranulation.

Overall, the tissue 4-MU degranulation assay would be beneficial supplement to studies evaluating inflammation and innate immunity. Advantages of this adaptation include the ability to evaluate degranulation in localized regions of tissue at a given timepoint, however, several important differences should be considered when comparing the modified and original assay designs. Firstly, in the originally described 4-MU assay, peripheral blood heterophils were isolated and concentrated to a known and standardized working concentration for all samples [23, 27]. When mucosal scrapings were collected for the modified assay, the number of heterophils present in a given sample were not quantified nor standardized and only released β-_D_-glucuronidase was quantified. Furthermore, intestinal samples are likely to have a variety of degranulating granulocytes present at any given time due to the constant flux of dietary antigens and resident microbial populations that can result in a chronic low-grade inflammatory status within the GIT [reviewed by 44]. As previously mentioned, mast cells, basophils, and eosinophils also contain β-_D_-glucuronidase in their granules, therefore, in addition to a basal level of degranulation, the modified assay quantifies total granulocyte degranulation [29–31]. Consequently, if any underlying conditions altered the overall health status of the bird resulting in changes in granulocyte activity, degranulation values may not be specifically reflective of treatment. Another important point of consideration is that resident bacteria, both commensals and opportunistic pathogens, release β-_D_-glucuronidase as a means to obtain glucuronic acid [40, 41]. Therefore, there is the potential for differences in the microbiotas of sampled birds to interfere with treatment-associated degranulation results through the production of β-_D_-glucuronidase, which authors attempted to mitigate by collecting samples into RPMI+1%ps.

An important aspect of assay design is inclusion of proper controls [75]. Since there was an expected basal level of degranulation, there was no perfect intestinal tissue negative control, instead lung tissue was collected as minimal degranulation was expected relative to the GIT. Heterophils do reside in bronchus-associated lymphoid tissue (BALT) of the lung, and respiratory pathogens and certain viral infections can lead to heterophil recruitment and degranulation in this tissue [76; reviewed by 77]. However, respiratory disease was not suspected in these studies and lung tissue appeared healthy upon collection. While RPMI+1%ps could also potentially serve as a negative control, it is important to consider that this would not accurately account for autofluorescence associated with tissue-derived supernatants. For example, flavins and extracellular matrix components such as elastin and collagen are known to produce a relatively high level of auto-fluorescence [reviewed by 78]. With regard to verifying that the 4-MU assay was in fact measuring the target enzyme, positive controls consisted of tissue samples stimulated with SOZ which has been shown to stimulate neutrophil and heterophil degranulation and result in increased β-_D_-glucuronidase [79–81]. All positive control values (Exp. 5–7) were in fact elevated relative to sample values strongly suggesting that 4-MU values were positively correlated with β-_D_-glucuronidase concentrations in samples.

A significant pitfall of the modified assay was a high degree of intraassay variability (Figs 3 and 4). A high degree of standard error observed within treatments may be due to factors including small sampling size and undefined biological variables within sample birds that may have influenced degranulation. Therefore, future iterations would benefit from sampling a larger number of birds to account for potential biological anomalies. The high degree of relative variability observed in some sample triplicates suggests that perhaps β-_D_-glucuronidase in supernatants was unevenly pipetted or acted inconsistently on the 4-MUG substrate. Additionally, due to the samples being of tissue origin, perhaps some replicates contained varying amounts of other auto-fluorescent components. Furthermore, in experiments 1–4, samples were fluorescently measured using an auto-gain setting whereas experiments 5–7 were measured at a gain setting of 70. The gain setting adjusts the amount of time a sample is excited. In the case of auto-gain, excitation time is optimized for each individual sample based on fluorescent signaling, whereas a set gain value exposes each sample to a constant excitation time [82]. Therefore, using a set gain, may be one means to reduce sample variability and control background. Additionally, using a set gain also resulted in a more consistent y-axis scale which allows for a more reliable comparison of results between experiments (Fig 3B–3D). For example, when comparing controls in experiments 5–7, C5, C6, and C7 had relatively similar levels of 4-MU which may be representative of baseline degranulation in healthy birds. Replication of these results could potentially provide a reference range for degranulation in the GIT of healthy birds in the future, although treatment responses may vary with respect to disease severity making establishment of treatment baseline values more challenging. Lastly, based on results, a strong unidirectional degree of inflammation, such as that documented for coccidial infection, may be necessary to observe treatment effects on granulocyte degranulation at this stage of assay development. Future iterations of this assay will aim to increase sensitivity for lower levels of inflammation and decrease intraassay standard errors. Additionally, supplementation of 4-MU assay results with histology would prove useful in assessing a correlation between observable populations of granulocytes and 4-MU values. Furthermore, supplemental markers such as IL-8 [83], oxidative burst [28], or granulocyte colony-stimulating factor [84] which are positively correlated with heterophil recruitment and degranulation could be beneficial in validating assay results and strengthening findings.

## Conclusions

While further refinement of the modified 4-MU assay is warranted, these results demonstrate successful adaptation of a previously defined cell culture degranulation assay for use with intestinal tissue. Experiments 1–3 presented here aimed to replicate the findings of Rodrigues et al. [32] and Wilson et al. [33] whom reported pioneer colonization by *in ovo* administered bacterial isolates resulted in alterations in responsiveness to inflammation. Functional predictions in IPA denoted increased inflammation but inhibited responsiveness to inflammation, such as the decreased movement of granulocytes. In the findings detailed here, neither *in ovo* inoculation with CF nor LAB resulted in significant alterations to 4-MU values at 10 days of age. A lack of responsiveness to inflammation in previous works [32, 33] could be attributed to the development of a tolerance response to the selected pioneer colonizing isolates, resulting in a blunted inflammatory response and immune cell function. Similarly, a lack of differences observed in 4-MU values in experiments 1–3 could be indicative of a similar response by 10 days of age. Due to a lack of noted pathogenicity, isolates may not have been capable of inducing severe inflammation resulting in quick resolution of any potentially mounted initial inflammatory response followed by possible development of tolerance. In further support of mild inflammatory response, minimal changes were captured by BWG which is generally reflective of significant inflammation and disease in poultry. To evaluate the efficacy of the 4-MU assay in more severe, prolonged instances of inflammation, a coccidial challenge model was used in experiments 4–7. Findings detail a previously undocumented phenomenon in the intestines of *Eimeria maxima* challenged broilers wherein measurable ileal granulocyte degranulation is significantly reduced relative to healthy, uninfected birds at time of peak lesions. Initial research into immune reactions to coccidiosis detail changes in circulating heterophil concentrations within hours of infection [49, 57], and therefore it is possible peak heterophil degranulation response was missed in these experiments. Consequently, 4-MU results from experiments 4–7 are likely the results of a high degree of mucosal sloughing from coccidial infection resulting in fewer granulocytes present in the tissue. Regardless of the mechanism responsible for the *Eimeria*-induced results, whether suppressed degranulation or fewer granulocytes present undergo degranulation, 4-MU values in experiments 4–7 demonstrate that the adapted assay is capable of detecting differences in the amount of β-_D_-glucuronidase activity in intestinal samples.

It is possible that in all experiments, sample collection was delayed with respect to primary response by granulocytes. Therefore, time course studies in future iterations would prove useful for determination of optimal sample collection timepoints in order to observe reliable and treatment-specific alterations in degranulation responses. At present, a given limitation is the lack of quantification of heterophils or other granulocytes in sampled tissues, thus posing the possibility that extraneous sources of β-_D_-glucuronidase influenced results, the primary source hypothesized to be resident bacteria. To resolve this, future iterations could culture assay supernatants for bacteria in order to determine the efficacy of the RPMI+1%ps in mitigating bacterial interference. A further weakness of the results described here is the high assay variability, which could primarily and firstly be addressed by including larger sample sizes and potentially more replicates per sample within the assay. Collectively, these findings provide foundational evidence that β-_D_-glucuronidase activity could be assessed in intestinal tissue to serve as a proxy for granulocyte degranulation. Through the described adapted 4-MU assay methodology, added real-time and site-specific insight into granulocyte degranulation in a given tissue can be achieved.

## Supporting information

S1 Data (XLSX)

S1 Raw data (XLSX)
